# The Electrolytic or Ionic Administration of Drugs

**Published:** 1909-06-26

**Authors:** 


### THE ELECTROLYTIC OR IONIC ADMINISTRATION OF DRUGS.

It is an elementary lesson in physics that teaches us that the passage of a continuous electric current through a solution of a salt causes the basic portion of the salt to go to the negative pole, and the acid portion to the positive pole; the amount of dissociated salt accumulated at either pole is directly proportional to the amount of current passed. The possibility of making use of this phenomenon in causing a given drug to accumulate and act precisely where it is required, either in the skin itself or in the parts beneath it, has long been obvious; but it is only of comparatively recent years that the method has been successfully employed to any extent.

Mr. Clague, in writing upon the subject recently, quotes some experiments made by Professor Leduc, of the University of Nantes, which illustrate in a remarkable way the extent to which the electrolytic method is effective in causing drugs to pass into the tissues through the unbroken skin.

Two rabbits were taken, an ear of one was strapped to an ear of the other by a pad of wet lint. To the outer ears were attached pads of lint moistened with solution of strychnine hydrochloride, and these were joined to a battery of cells. The rabbit connected positively died promptly, the ions of the base—strychnine—having been driven into that animal electrolytically. The other rabbit, through which precisely the same electric current had been flowing, remained alive until the current was reversed, when it was poisoned like the first. A second experiment was made with the same technique, but with cyanide of potassium instead of strychnine hydrochloride. In this case the negatively connected rabbit died, the poisonous ions being the acid ones of hydrocyanic acid. On reversing the current the second rabbit died.

Many other interesting points will be found in Mr. Clague's paper (*Pharmaceut. Journ.*, 1908, p. 346).

The use of electrolysis has long been made in the removal of superfluous hairs, nævi, and so forth. The curative results are due to the saline fluids of the body becoming electrolysed. A platinum or steel needle is inserted into each hair sheath and connected to the negative pole of a battery of about four cells, and the electric current is allowed to pass for about five seconds. This causes the accumulation locally of a small but definite quantity of caustic soda, the vital part of the hair being destroyed by the latter.

Lithium, iodides, or salicylates are all capable of being introduced into the body by the electrolytic method, and when action is required locally—in a gouty joint, for instance—the direction in which the current has to flow through the patient will be the opposite when bases such as lithium are to be introduced, to what it is when acids such as iodide or salicylate are to be driven in.

It has frequently been observed that small doses of magnesium sulphate have caused the disappearance of warts. The electrolytic method has made it possible to remove warts readily, magnesium salts being employed in connection with the electric current. The results are said to be excellent, and there is no scarring such as that which results from knife or caustic. The *x*-rays remove warts equally without scar, but the *x*-rays require more special apparatus than does electrolysis, so that many more cases can be treated by the latter than by the former process. The negative current and sodium salicylate afford great relief to corns. The positive wire in contact with the patient should be one which does not electrolyse under the conditions employed; iron, copper, and silver are consequently not good; platinum or aluminium are preferable. It is important to keep to simple salts as far as possible.

Considerable differences exist as regards individual sensitiveness to the electric current: some patients complaining little if at all, others, on the other hand, flinching even at the idea of the weakest currents. As a rule, however, this is not a source of great difficulty, for the amount of current required is not very large.

